# Daily lifestyle behaviors and risks of sarcopenia among older adults

**DOI:** 10.1186/s13690-020-00498-9

**Published:** 2020-11-10

**Authors:** Pei-Lin Tzeng, Chien-Yu Lin, Ting-Fu Lai, Wan-Chi Huang, Evonne Pien, Ming-Chun Hsueh, Kun-Pei Lin, Jong-Hwan Park, Yung Liao

**Affiliations:** 1 Department of Health Promotion and Health Education, National Taiwan Normal University, Taipei, Taiwan; 2 Department of Geriatrics and Gerontology, National Taiwan University Hospital, Taipei, Taiwan; 3 Graduate School of Sport Sciences, Waseda University, Tokorozawa City, Japan; 4 Department of Psychology, University of California, San Diego, USA; 5 Graduate Institute of Sport Pedagogy, University of Taipei, Taipei, Taiwan; 6 Department of Internal Medicine, National Taiwan University Hospital, Taipei, Taiwan; 7 Health Convergence Medicine Laboratory, Biomedical Research Institute, Pusan National University Hospital, Busan, South Korea; 8 Faculty of Sport Sciences, Waseda University, Tokorozawa City, Japan

**Keywords:** Behavior change, Lifestyle intervention, Health promotion

## Abstract

**Background:**

Lifestyle behaviors are modifiable factors that can provide information for designing intervention strategies for sarcopenia. The present study aimed to identify the relationships between a range of daily lifestyle behaviors and sarcopenia risks among older adults.

**Methods:**

A nationwide telephone-based survey targeting older adults (≥65 years) was performed in Taiwan. Data based on self-reported daily lifestyle behaviors (food selection, physical activity, sitting time, and sleep duration), the presence or absence of sarcopenia (measured by SARC-F), and personal characteristics were obtained. Binary logistic regression models were applied.

**Results:**

A total of 1068 older adults participated in this survey. In the adjusted model, older adults who selected unbalanced foods (odds ratio [OR] = 1.93, 95% confidence interval [CI] = 1.12–3.34), engaged in insufficient physical activity (OR = 5.14, 95% CI = 3.04–8.70), and sat for longer periods of time (OR = 1.98, 95% CI = 1.09–3.59) were more likely to have higher risks of sarcopenia. No significant association was observed for sleep duration.

**Conclusions:**

The results of this study highlight that, among health behaviors, an unbalanced food selection (six nutrients), not meeting physical activity recommendations (150 min/week), and a higher sitting time (≥7 h/day) were risk factors for sarcopenia among older adults. Intervention programs for sarcopenia prevention in older adults should focus on promoting balanced food selection, sufficient physical activity, and reduced sitting time.

## Background

Sarcopenia is a geriatric syndrome caused by the progressive loss of skeletal muscle mass and function with age [1]. It is associated with increased risks of falls, disability, poor quality of life, and premature death [2, 3]. Despite the aforementioned adverse health outcomes, recent systematic reviews have revealed sarcopenia to be present in approximately 10% of the older adults worldwide [4, 5], which could lead to economic and societal burdens [6, 7]. With the rapidly growing number of older adults [8], especially in Eastern Asia, where this rate is the highest [9], sarcopenia is also expected to increase significantly [10]. Therefore, there is a need to design effective sarcopenia prevention programs or to develop strategies to prevent sarcopenia that can be incorporated in the daily lives of older adults [11].

The risk factors of lifestyle health behaviors for sarcopenia are controllable and modifiable on a daily basis and are also markedly related to the progression of the most common non-communicable diseases [12]. Therefore, it is important to investigate specific lifestyle behaviors in the everyday lives of older adults to prevent sarcopenia. Systematic reviews have identified some lifestyle behaviors such as physical activity [13, 14] and sleep duration [15] to be associated with higher sarcopenia risks. Moreover, previous research has also investigated the association between specific nutrients [16, 17], rather than overall food selection, and sarcopenia. It is of value to know whether adherence to the current Taiwanese dietary guidelines can play a protective role against sarcopenia in older adults. Furthermore, a number of studies have targeted sedentary behavior, which may occupy considerable amounts of their time each day [18], and is a novel risk behavior for poor health in older adults [19]. Although there were some studies that examined the linear [20, 21] and non-linear associations [22] between sedentary behavior and sarcopenia among the older population, a recent meta-analysis has reported the novel evidence of a cutoff point of 7 h/day for self-reported measures of sedentary time for mortality [23]. To the best of our knowledge, no previous research has examined the cutoff point for sarcopenia risk with this new evidence; therefore, further investigation should be conducted with this updated evidence.

Taiwan provides a unique setting to investigate lifestyle factors and sarcopenia. This setting is representative of Asian countries with respect to its specific diet- and activity-related cultures. For example, a typical Asian meal usually comprises rice as a staple with several side dishes. Furthermore, some specific physical activities such as tai chi and qigong are common in the older adults living in Asian countries. Over the last four decades, the older population in Taiwan has more than tripled, with the percentage dramatically increasing from 4.4 to 16% [24]. However, to our knowledge, no study has examined the associations between a range of daily lifestyle behaviors and sarcopenia risks in Taiwan. Such evidence based on the local context is particularly critical for intervention designers and health-promotion practitioners. We hypothesized that, among older Taiwanese adults, each unhealthy lifestyle behavior was associated with an increased risk of sarcopenia. Therefore, this study examined the associations of four daily lifestyle behaviors with the risks of sarcopenia in a sample of older Taiwanese adults.

## Methods

### Participants and procedures

From October 2019 to January 2020, a cross-sectional telephone-based investigation targeting Taiwanese older adults (≥65 years) was conducted. The sampling was conducted by a random-digit-dialing telephone-based survey using the national household telephone directory with landline telephone numbers. The potential target population comprised 3,607,127 older adults in December 2019 [25]. We used a two-stage sampling procedure to obtain a nationwide representative sample whose basic characteristics matched those of the older adult population in Taiwan. The first stage geographically stratified Taiwan into four areas (eastern, southern, western, and northern). Further, in the second stage, the participants were randomly chosen according to sex (men or women) and age group (65–74 or over 75 years). All the telephone calls were performed by a telephone research service company. In each telephonic interview, a standardized and structured questionnaire was administered by a well-trained interviewer who had received at least 8 h of training prior to the survey. The interviewers were trained to identify the participants’ ability to comprehend and answer the questions. If any participant could not understand specific questions or answer the questions logically even after receiving an explanation, the survey was suspended. Furthermore, technical (e.g., flicker noise) and other conditions (e.g., interruptions) led to some incomplete surveys, which were excluded from our analysis. In the survey duration, a total of 2352 older adults were interviewed, 1068 of whom completed the survey (response rate: 45.4%). No incentives were provided. Verbal informed consent was obtained at the beginning of each phone survey. All the procedures used in this study were reviewed and confirmed by the Research Ethics Committee of National Taiwan Normal University (REC number: 201706HM020).

### Sarcopenia

Sarcopenia was identified using SARC-F, a quick screening tool for sarcopenia that is widely used in several countries [26–28]. The SARC-F score has been validated to be inversely associated with the grip strength and the percentage of skeletal muscle mass in Taiwanese older adults [29]. The SARC-F questionnaire included 5 items: (1) sluggishness; (2) assistance in walking; (3) rising from a chair; (4) climbing stairs; and, (5) falls. Detailed descriptions of these five items have been reported previously [29]. Each item was scored from 0 to 2, and the scores were summed up to calculate the total score. Scores of 4 or higher were categorized under sarcopenia [26].

### Daily lifestyle behaviors

This study examined the associations of four different daily lifestyle behaviors with sarcopenia risks.

#### Food selection

The participants were requested to answer the question, “Do you have a habit of the balanced intake of the six categories of food according to the Taiwanese dietary guidelines?” as yes (balanced) or no (unbalanced). The six categories of food included were “whole grains,” “vegetables,” “fruit,” “dairy products,” “legumes, fish, eggs, and meat,” and “oils, fats, nuts, and seeds.” If the participants enquired about the definition of a balanced diet, the interviewers were trained to provide an instruction along with the question based on the dietary guidelines [30]. The yes/no question has also been utilized in previous investigations of older Taiwanese adults [31–33].

#### Physical activity

Physical activity was assessed by the International Physical Activity Questionnaire––short version (IPAQ-SV). This questionnaire was widely used in surveys of older populations across countries [34–36]. Its test-retest reliability was 0.78 and the criterion validity (compared with accelerometers) was 0.31–0.41 [37]. This questionnaire comprised seven items that measured the frequency and duration of walking behavior, moderate-intensity activity, and vigorous-intensity activity. The total weekly minutes of each activity category were first summed and categorized as sufficient and insufficient physical activity levels based on the physical activity guideline of 150 min or more per week [38].

#### Sitting time

The sitting time was also measured by the IPAQ-SV. The participants were asked, “In the last seven days, how much time did you usually spend sitting in a day?” According to a previous review of sedentary time cutoff points, a higher sitting time was defined as ≥7 h/day [23, 39]. Accordingly, the responses in the present study were categorized as “high” (sitting ≥7 h/day) and “low” (< 7 h/day).

#### Sleep duration

Self-reported sleep duration was measured from the responses to the following question, “In the past month, how many hours did you actually sleep on an average night?”, which was similar to that asked in a previous study [40] and a question in the Pittsburgh Sleep Quality Index questionnaire [41]. The overall sleep duration was categorized into “suggested sleep” (sleep duration of 7–8 h/day), recommended by National Sleep Foundation [42] and “shorter or longer sleep” (sleep duration of < 7 or ≥ 8 h/day) [43, 44].

### Covariates

The self-reported demographics were collected through a standardized questionnaire and were included as covariates in the analyses. Demographics including sex, age group (65–74 or 75+ years), residential geographic areas (eastern, southern, middle, and northern), education (elementary or below, middle or high school, and university or higher), marital status (married and unmarried/divorced/widowed), employment (full-time and not full-time), living status (alone and not alone), height, and weight were reported. Body mass index was defined as the weight (in kilograms) divided by the square of height (in meters) and the cutoffs for the different categories (underweight, normal, overweight, or obese) used were the standards provided by the Ministry of Health and Welfare of Taiwan [45]. The participants were categorized as underweight (< 18.5 kg/m^2^), normal weight (18.5–24 kg/m^2^), overweight (> 24–30 kg/m^2^), and obese (> 30 kg/m^2^).

### Statistical analyses

We used chi-square tests to examine the differences in demographic characteristics and lifestyle factors between the two sarcopenia risk groups. Further analyses using binary logistic regression models estimated the odds ratios (ORs) and 95% confidence intervals (CIs) of the lifestyle factors for the high-risk sarcopenia group (i.e., compared with the low-risk group), before and after adjusting for covariates. All analyses were conducted using IBM SPSS Statistics for Windows, version 23.0 (IBM Corp., Armonk, NY, 2011), and the level of significance was set at *p* < 0.05.

## Results

The analyses included a total of 1068 older adults among the individuals over 65 years of age in Taiwan during the study period. Approximately half of the study population comprised men (*n* = 505, 47.3%). The mean age was 72.1 years (range, 65–92 years), and the prevalence of sarcopenia was 7.3% (*n* = 78) (Table 1). Compared with individuals without sarcopenia, those with sarcopenia were more likely to be female, be older, have a lower educational level, be single, lack a full-time job, live alone, have an unhealthy body weight, report the selection of unbalanced foods, engage in insufficient physical activity, sit for less than 7 h/day, and have shorter or longer sleep durations (Table 1).

**Table 1:** Characteristics of the study sample (*n* = 1068)

Studied characteristics	Non-sarcopenia		Sarcopenia		χ2	Degrees of freedom	*p*-value
	n	%	n	%			
Sex					14.00	1	< 0.001
Men	484	48.9%	21	26.9%			
Women	506	51.1%	57	73.1%			
Age group (years)					15.16	1	< 0.001
65–74	693	70.0%	38	48.7%			
75+	297	30.0%	40	51.3%			
Residential geographic areas					6.19	3	0.103
Northern	425	42.9%	23	29.5%			
Middle	238	24.0%	20	25.6%			
Southern	272	27.5%	29	37.2%			
Eastern and island	55	5.6%	6	7.7%			
Education					20.07	2	< 0.001
Elementary or below	357	36.1%	48	61.5%			
Middle or high school	374	37.8%	19	24.4%			
University or higher	259	26.2%	11	14.1%			
Marital status					12.53	1	< 0.001
Married	781	78.9%	48	61.5%			
Unmarried/divorced/widowed	209	21.1%	30	38.5%			
Employment					8.84	1	0.003
Full-time	124	12.5%	1	1.3%			
Not full-time	866	87.5%	77	98.7%			
Living status					5.47	1	0.019
Alone	86	8.7%	13	16.7%			
Not alone	904	91.3%	65	83.3%			
Body mass index					12.16	3	0.007
Underweight (< 18.5 kg/m^2^)	32	3.2%	5	6.4%			
Normal weight (18.5–24 kg/m^2^)	496	50.1%	32	41.0%			
Overweight (> 24–30 kg/m^2^)	312	31.5%	19	24.4%			
Obese (> 30 kg/m^2^)	150	15.2%	22	28.2%			
Food selection					10.78	1	0.001
Balanced	791	79.9%	50	64.1%			
Unbalanced	199	20.1%	28	35.9%			
Physical activity					74.55	1	< 0.001
Sufficient	861	87.0%	39	50.0%			
Insufficient	129	13.0%	39	50.0%			
Sitting time					10.08	1	0.002
Low (< 7 h/day)	145	14.6%	22	28.2%			
High (≥7 h/day)	845	85.4%	56	71.8%			
Sleep duration					4.57	1	0.033
Suggested (7–8 h/day)	290	29.3%	14	17.9%			
Shorter or longer (< 7 or > 8 h/day)	700	70.7%	64	82.1%			

In the unadjusted model, all the lifestyle factors investigated were associated with sarcopenia risk (Table 2). Overall, older adults who selected unbalanced foods, engaged in insufficient physical activity, sat for ≥7 h/day, and slept less or more had higher risks of sarcopenia. After adjusting for all the covariates, the strengths of most of the associations were attenuated. However, there was evidence for the associations of sarcopenia risk with selecting unbalanced foods (OR = 1.93, 95% CI = 1.12–3.34), insufficient physical activity (OR = 5.14, 95% CI = 3.04–8.70), and prolonged sitting time (OR = 1.98, 95% CI = 1.09–3.59). No significant association was observed between sarcopenia risk and sleep duration (OR = 1.26, 95% CI = 0.74–2.16).

**Table 2:** Odds ratios and 95% confidence intervals of daily lifestyle factors associated with sarcopenia risk (*n* = 1068)

Studied characteristics	Unadjusted			Adjusted^a^		
	OR	(95% CI)		OR	(95% CI)	
Food selection						
Balanced	1.00	–	–	1.00	–	–
Unbalanced	**2.23**	**(1.37**	**, 3.63)**	**1.93**	**(1.12**	**, 3.34)**
Physical activity						
Sufficient	1.00	–	–	1.00	–	–
Insufficient	**6.67**	**(4.13**	**, 10.80)**	**5.14**	**(3.04**	**, 8.70)**
Sitting time						
Low (< 7 h/day)	1.00	–	–	1.00	–	–
High (≥7 h/day)	**2.29**	**(1.36**	**, 3.87)**	**1.98**	**(1.09**	**, 3.59)**
Sleep duration						
Suggested (7–8 h/day)	1.00	–	–	1.00	–	–
Shorter or longer (< 7 or > 8 h/day)	**1.89**	**(1.05**	**, 3.43)**	1.26	(0.74	, 2.16)

^a^The models are adjusted for sex, age group, residential geographic areas, education, marital status, employment, living status, body mass index, and all the other characteristics in the Table. *OR* Odds ratio; *CI* Confidence interval

## Discussion

Given the lack of evidence regarding the modifiable risk factors for sarcopenia in Taiwan, this nationwide survey examined a range of lifestyle behaviors and their associations with sarcopenia risks in a representative sample of older Taiwanese adults. Consistent with previous findings [17, 46], the key findings of the present study indicated that, after controlling for potential covariates, older adults who selected unbalanced foods, did not achieve physical activity recommendations, and engaged in prolonged sitting times were more likely to be at a risk of sarcopenia. However, sleep duration was not related to sarcopenia risk, which is inconsistent with the previously reported findings [15]. The lack of an association between sleep duration and sarcopenia may be attributable to the small sample size. Future studies using prospective designs or meta-analyses pooling data to include a large sample size to test the associations of diverse lifestyle factors with sarcopenia are suggested. Although the associations between these daily lifestyle behaviors and sarcopenia are already known, our findings provide important local evidence with advanced cutoff points of sedentary risks and suggest that behavioral change-related interventions including education, training, and enablement should prioritize food selection, physical activity, and sitting time.

Our findings provide evidence that the older adults who selected a balanced diet following the Taiwanese dietary guideline (six categories of food) [30] were less likely to be at a risk of sarcopenia. However, this finding was inconsistent with that of a previous study in Western countries that reported that adherence to the dietary pattern was not related to sarcopenia in older adults [47]. This difference may be attributable to the variations in dietary patterns across countries. The dietary pattern in Taiwan is characterized by grains, dairy products, vegetables and fruits, meat, and fats whereas that in Western countries often features a high consumption of soy, sugar, and fast foods. A balanced food selection based on the Taiwanese guidelines may enable older adults to consume sufficient nutrients (e.g., protein, amino acids, and vitamins D and E) as a part of their daily food consumption [48].

Systematic reviews have shown that achieving the recommended levels of physical activity is an effective protective strategy against sarcopenia and has a positive impact on muscle mass/strength and physical capacities in older adults [13, 14]. The benefits of physical activity are attributed to the activation of muscle stem cells by physical activity through increased protein synthesis or new satellite cell fusions [49]. Therefore, achieving the current guideline of 150 min/week [50] is critical for sarcopenia prevention in older adults. Moreover, previous reviews and studies have indicated a lack of evidence on the relationship between food selection and sarcopenia risk in older adults, highlighting the significance of implementing lifestyle behaviors by conforming to dietary and physical activity guidelines for older adults.

Another important finding was that the older adults with daily sitting times ≥7 h were more likely to have sarcopenia risks; this was similar to previous findings that showed that the risk of sarcopenia increased by 6–33% for each 1-h increase in sedentary behavior per day [21, 22]. However, previous studies using different risk cutoffs of sedentary behavior reported that the sarcopenia risk of older adults sitting for 11 h/day or more was twice of those sitting for less than 4 h/day [22]. The study highlighted, with advanced evidence, the sarcopenia risk of sitting for more than 7 h/day in older adults. These positive associations may be explained by the relationship between a longer sitting time and increased levels of inflammatory markers and the lack of skeletal muscular contractions, which may contribute to an accelerated loss of muscle mass and strength [21, 51]. Moreover, lower muscle mass and strength were associated with lower limb dysfunction [52, 53], which leads to difficulties in performing daily errands. Therefore, these findings suggest the importance of encouraging older adults to limit their sitting time to less than 7 h/day for sarcopenia prevention.

This study had several limitations. First, this study used self-reported measures of lifestyle behaviors and risks of sarcopenia, which may lead to recall bias in older adults. Moreover, physical activity and sitting time were not assessed by different domains (i.e., leisure, transportation), which are related to different health outcomes [34, 54]. In addition, food selection was measured by a single question on general dietary consumption rather than questions on specific dietary constituents such as vitamin D and E, proteins, and amino acids, which are associated with sarcopenia risk [16, 17]. Despite these limitations, these self-reported measures have been shown to be valid and suitable for use in nationwide epidemiological surveys. Second, owing to its cross-sectional design, the present study could not determine causality. Finally, this study also had a limited representative sample size since the survey was conducted through phone calls. Thus, individuals without a household telephone (approximately 10.4% in 2018) could not be reached [55]. Moreover, during the telephonic survey, there is a potential risk of social desirability bias while answering the questions asked by interviewers.

## Conclusions

The results of this study highlight that, among a range of lifestyle behaviors, selection of unbalanced foods (failure to eat six categories of food), not meeting physical activity recommendations (150 min/week), and prolonged sitting times (≥7 h/day) were risk factors for sarcopenia among older Taiwanese adults. The study also highlights the sarcopenia risk of sitting for more than 7 h among the older population, with advanced evidence. Intervention programs for sarcopenia prevention in older adults should focus on promoting the selection of balanced foods, sufficient physical activity, and reduced sitting time.

## Data Availability

The data used during the present study are available from the corresponding author upon reasonable request.

## Abbreviations

IPAQ-SV: International physical activity questionnaire – short version
OR: Odds ratio
CI: Confidence interval

## References

[1] Santilli V, Bernetti A, Mangone M, Paoloni M (2014). Clinical definition of sarcopenia. Clin Cases Miner Bone Metab.

[2] Veronese N, Demurtas J, Soysal P, Smith L, Torbahn G, Schoene D, Schwingshackl L, Sieber C, Bauer J, Cesari M (2019). Sarcopenia and health-related outcomes: an umbrella review of observational studies. Eur Geriatr Med.

[3] Beaudart C, Zaaria M, Pasleau F, Reginster J-Y, Bruyère O (2017). Health outcomes of sarcopenia: a systematic review and Meta-analysis. PLoS One.

[4] Mayhew AJ, Amog K, Phillips S, Parise G, McNicholas PD, de Souza RJ, Thabane L, Raina P (2019). The prevalence of sarcopenia in community-dwelling older adults, an exploration of differences between studies and within definitions: a systematic review and meta-analyses. Age Ageing.

[5] Shafiee G, Keshtkar A, Soltani A, Ahadi Z, Larijani B, Heshmat R (2017). Prevalence of sarcopenia in the world: a systematic review and meta- analysis of general population studies. J Diabetes Metab Disord.

[6] Beaudart C, Rizzoli R, Bruyère O, Reginster J-Y, Biver E (2014). Sarcopenia: burden and challenges for public health. Arch Public Health.

[7] Bruyère O, Beaudart C, Ethgen O, Reginster J-Y, Locquet M (2019). The health economics burden of sarcopenia: a systematic review. Maturitas.

[8] United Nations: Ageing. Available online: https://www.un.org/en/sections/issues-depth/ageing/. (Accessed 5 Nov 2020).

[9] United Nations, Department of Economic and Social Affairs, Population Division: **World Population Ageing** 2019**: Highlights**. In*.*; 2019.

[10] Ethgen O, Beaudart C, Buckinx F, Bruyère O, Reginster JY (2017). The future prevalence of sarcopenia in Europe: a claim for public health action. Calcif Tissue Int.

[11] Cruz-Jentoft AJ, Sayer AA (2019). Sarcopenia. Lancet.

[12] Byrne DW, Rolando LA, Aliyu MH, McGown PW, Connor LR, Awalt BM, Holmes MC, Wang L, Yarbrough MI (2016). Modifiable healthy lifestyle behaviors: 10-year health outcomes from a health promotion program. Am J Prev Med.

[13] Lee SY, Tung HH, Liu CY, Chen LK (2018). Physical activity and sarcopenia in the geriatric population: a systematic review. J Am Med Dir Assoc.

[14] Beaudart C, Dawson A, Shaw SC, Harvey NC, Kanis JA, Binkley N, Reginster JY, Chapurlat R, Chan DC, Bruyere O (2017). Nutrition and physical activity in the prevention and treatment of sarcopenia: systematic review. Osteoporos Int.

[15] Pourmotabbed A, Ghaedi E, Babaei A, Mohammadi H, Khazaie H, Jalili C, Symonds ME, Moradi S, Miraghajani M. Sleep duration and sarcopenia risk: a systematic review and dose-response meta-analysis. Sleep Breath. 2019. 10.1007/s11325-019-01965-6 31832982

[16] Bloom I, Shand C, Cooper C, Robinson S, Baird J. Diet quality and sarcopenia in older adults: a systematic review. Nutrients. 2018;10(3):308. 10.3390/nu10030308 PMC5872726 29510572

[17] Shaw SC, Dennison EM, Cooper C (2017). Epidemiology of sarcopenia: determinants throughout the Lifecourse. Calcif Tissue Int.

[18] Harvey JA, Chastin SF, Skelton DA (2015). How sedentary are older people? A systematic review of the amount of sedentary behavior. J Aging Phys Act.

[19] de Rezende LFM, Rodrigues Lopes M, Rey-López JP, Matsudo VKR, OdC L (2014). Sedentary behavior and health outcomes: an overview of systematic reviews. PLoS One.

[20] Scott D, Johansson J, Gandham A, Ebeling PR, Nordstrom P, Nordstrom A. Associations of accelerometer-determined physical activity and sedentary behavior with sarcopenia and incident falls over 12 months in community-dwelling Swedish older adults. J Sport Health Sci. 2020. 10.1016/j.jshs.2020.01.006 PMC8500807 34088651

[21] Gianoudis J, Bailey CA, Daly RM (2015). Associations between sedentary behaviour and body composition, muscle function and sarcopenia in community-dwelling older adults. Osteoporos Int.

[22] Smith L, Tully M, Jacob L, Blackburn N, Adlakha D, Caserotti P, Soysal P, Veronese N, Sánchez GFL, Vancampfort D, Koyanagi A. The Association Between Sedentary Behavior and Sarcopenia Among Adults Aged ≥65 Years in Low- and Middle-Income Countries. Int J Environ Res Public Health. 2020;17(5):1708. 10.3390/ijerph17051708 PMC7084450 32151034

[23] Ku PW, Steptoe A, Liao Y, Hsueh MC, Chen LJ (2018). A cut-off of daily sedentary time and all-cause mortality in adults: a meta-regression analysis involving more than 1 million participants. BMC Med.

[24] National Development Council, Republic of China (TAIWAN). Estimated Future Population: Aging Indicators. Available online: https://pop-proj.ndc.gov.tw/chart.aspx?c=10&uid=66&pid=60. (Accessed 5 Nov 2020).

[25] Ministry of the Interior Population, Republic of China (TAIWAN): Monthly Bulletin of Interior, Statistics by Age of 0-14, 15-64,65+ and by 6-year Age Group. Available online: https://www.moi.gov.tw/files/site_stuff/321/1/month/month_en.html. (Accessed 5 Nov 2020).

[26] Malmstrom TK, Morley JE (2013). SARC-F: a simple questionnaire to rapidly diagnose sarcopenia. J Am Med Dir Assoc.

[27] Kim S, Kim M, Won CW (2018). Validation of the Korean version of the SARC-F questionnaire to assess sarcopenia: Korean frailty and aging cohort study. J Am Med Dir Assoc.

[28] Mohd Nawi SN, Khow KS, Lim WS, Yu SC (2019). Screening tools for sarcopenia in community-dwellers: a scoping review. Ann Acad Med Singap.

[29] Wu T, Liaw C, Chen F, Kuo K, Chie W, Yang R (2017). Sarcopenia screened with SARC-F questionnaire is associated with quality of life and mortality. Innov Aging.

[30] Administration HP, Health Promotion Administration (2018). New Guidelines on Daily Nutrition.

[31] Chang CF, Lin MH, Wang J, Fan JY, Chou LN, Chen MY (2013). The relationship between geriatric depression and health-promoting behaviors among community-dwelling seniors. J Nurs Res.

[32] Wang J, Lee C-M, Chang C-F, Jane S-W, Chen M-Y (2015). The development and psychometric testing of the geriatric health promotion scale. J Nurs Res.

[33] Hsueh MC, Rutherford R, Huang YH, Chang Chien HY, Chang CH, Park JH, Liao Y. Are Older Adults without a Healthy Diet Less Physically Active and More Sedentary? Nutrients. 2019;11(5):1119. 10.3390/nu11051119 PMC6566680 31137459

[34] Hsueh M-C, Liao Y, Chang S-H (2016). Associations of Total and domain-specific sedentary time with type 2 diabetes in Taiwanese older adults. J Epidemiol.

[35] Nemoto Y, Sato S, Takahashi M, Takeda N, Matsushita M, Kitabatake Y, Maruo K, Arao T (2018). The association of single and combined factors of sedentary behavior and physical activity with subjective cognitive complaints among community-dwelling older adults: cross-sectional study. PLoS One.

[36] Kim HS, Harada K, Miyashita M, Lee EA, Park JK, Nakamura Y (2011). Use of senior center and the health-related quality of life in Korean older adults. J Prev Med Public Health.

[37] Liou YM, Jwo CJ, Yao KG, Chiang LC, Huang LH (2008). Selection of appropriate Chinese terms to represent intensity and types of physical activity terms for use in the Taiwan version of IPAQ. J Nurs Res.

[38] Nelson ME, Rejeski WJ, Blair SN, Duncan PW, Judge JO, King AC, Macera CA, Castaneda-Sceppa C (2007). Physical activity and public health in older adults: recommendation from the American College of Sports Medicine and the American Heart Association. Med Sci Sports Exerc.

[39] Chau JY, Grunseit AC, Chey T, Stamatakis E, Brown WJ, Matthews CE, Bauman AE, van der Ploeg HP (2013). Daily sitting time and all-cause mortality: a meta-analysis. PLoS One.

[40] Stenholm S, Kronholm E, Bandinelli S, Guralnik JM, Ferrucci L (2011). Self-reported sleep duration and time in bed as predictors of physical function decline: results from the InCHIANTI study. Sleep.

[41] Buysse DJ, Reynolds CF, Monk TH, Berman SR, Kupfer DJ (1989). The Pittsburgh sleep quality index: a new instrument for psychiatric practice and research. Psychiatry Res.

[42] Hirshkowitz M, Whiton K, Albert SM, Alessi C, Bruni O, DonCarlos L, Hazen N, Herman J, Katz ES, Kheirandish-Gozal L (2015). National Sleep Foundation's sleep time duration recommendations: methodology and results summary. Sleep Health.

[43] Watson NF, Badr MS, Belenky G, Bliwise DL, Buxton OM, Buysse D, Dinges DF, Gangwisch J, Grandner MA, Kushida C (2015). Recommended amount of sleep for a healthy adult: a joint consensus statement of the American Academy of sleep medicine and Sleep Research Society. Sleep.

[44] Gangwisch JE, Heymsfield SB, Boden-Albala B, Buijs RM, Kreier F, Opler MG, Pickering TG, Rundle AG, Zammit GK, Malaspina D (2008). Sleep duration associated with mortality in elderly, but not middle-aged, adults in a large US sample. Sleep.

[45] Health Promotion Administration, Republic of China (TAIWAN): The measurement of BMI. Available online: https://health99.hpa.gov.tw/plus/onlineQuiz/bmi. (Accessed 5 Nov 2020).

[46] Rom O, Kaisari S, Aizenbud D, Reznick AZ (2012). Lifestyle and sarcopenia-etiology, prevention, and treatment. Rambam Maimonides Med J.

[47] Hashemi R, Motlagh AD, Heshmat R, Esmaillzadeh A, Payab M, Yousefinia M, Siassi F, Pasalar P, Baygi F (2015). Diet and its relationship to sarcopenia in community dwelling Iranian elderly: a cross sectional study. Nutrition.

[48] Loprinzi PD, Loenneke JP, Hamilton DL (2017). Leisure time sedentary behavior, physical activity and frequency of protein consumption on lower extremity strength and lean mass. Eur J Clin Nutr.

[49] Ceccarelli G, Benedetti L, Arcari ML, Carubbi C, Galli D (2017). Muscle stem cell and physical activity: what point is the debate at?. Open Med (Wars).

[50] World Health Organization: Global recommendations on physical activity for health. In.; 2010: 1–58. 26180873

[51] Schaap LA, Pluijm SMF, Deeg DJH, Harris TB, Kritchevsky SB, Newman AB, Colbert LH, Pahor M, Rubin SM, Tylavsky FA (2009). Higher inflammatory marker levels in older persons: associations with 5-year change in muscle mass and muscle strength. J Gerontol A Biol Sci Med Sci.

[52] Falsarella GR, Coimbra IB, Barcelos CC, Iartelli I, Montedori KT, Santos MN, Neri AL, Coimbra AM (2014). Influence of muscle mass and bone mass on the mobility of elderly women: an observational study. BMC Geriatr.

[53] Reid KF, Naumova EN, Carabello RJ, Phillips EM, Fielding RA (2008). Lower extremity muscle mass predicts functional performance in mobility-limited elders. J Nutr Health Aging.

[54] Liao Y, Tsai HH, Wang H-S, Lin C-P, Wu MC, Chen J-F. Travel mode, transportation-related physical activity, and risk of overweight in Taiwanese adults. J Transp Health. 2016;3(2):220-25.

[55] Directorate General of Budget, Accounting and Statistics, Republic of China (TAIWAN): Report on the Survey of Family Income and Expendture. Available online: https://www.dgbas.gov.tw/ct.asp?xItem=19882&CtNode=3241&mp=1. (Accessed 17 Feb 2020).

